# Absence of Epidermal Antibodies in Stevens–Johnson Syndrome/Toxic Epidermal Necrolysis Patients but Beware of Single Positive Results

**DOI:** 10.1155/2024/5504462

**Published:** 2024-05-20

**Authors:** Gilles F. H. Diercks, Joost M. Meijer, Maria C. Bolling, Sonja M. H. J. Scholtens-Jaegers, Jeroen Bremer, Barbara Horvath

**Affiliations:** ^1^University of Groningen, University Medical Center Groningen, Department of Dermatology, Center of Blistering Diseases, European Reference Networks-Skin Member, Groningen, Netherlands; ^2^European Reference Networks (ERN)—SKIN Center, Paris, France; ^3^Martini Hospital, Burn Centre Groningen, Groningen, Netherlands

## Abstract

**Background:**

Stevens–Johnson syndrome (SJS) and toxic epidermal necrolysis (TEN) are rare and potentially life-threatening mucocutaneous blistering diseases that clinically can resemble autoimmune bullous diseases. Moreover, it has been shown that autoantibodies against epidermal proteins are present in SJS/TEN.

**Objectives:**

To establish the presence of antibodies against desmosomal and hemidesmosomal proteins in confirmed SJS/TEN patients.

**Methods:**

Serum of SJS/TEN patients diagnosed based on clinical criteria, e.g., epidermal detachment with erosions and severe mucosal lesions, (suspicion of) a culprit drug, and matching histologic results was evaluated by various techniques, e.g., indirect immunofluorescence on monkey esophagus, salt split skin and rat bladder, immunoblotting (IB) and immunoprecipitation (IP), ELISAs against desmogleins and BP180, keratinocyte footprint assay, and keratinocyte binding assay.

**Results:**

A total of 28 patients were included in this study, 15 men and 13 women with a mean age of 56 years. In most patients, none of the serological tests were positive. In two patients, an elevated DSG3 titer was found suspicious for pemphigus vulgaris. Three patients had elevated NC16a titers, suggesting bullous pemphigoid. However, in all these patients, no other tests were positive and in these patients, the biopsy for direct immunofluorescence showed no evidence for an autoimmune bullous disease. Three patients showed reactivity against rat bladder rat bladder; these were, however, completely negative for A2ML1, envoplakin, and periplakin in the IB as well as the IP.

**Conclusions:**

Serological analysis for desmosomal and hemidesmosomal antibodies is reliable to rule an autoimmune bullous disease in patients with suspected SJS/TEN. However, one should not rely on one single test method since false positive results can occur. Moreover, this study also makes it less plausible that antibodies against desmosomal and/or hemidesmosomal components are involved in the pathogenesis of SJS/TEN.

## 1. Introduction

Stevens–Johnson syndrome (SJS) and toxic epidermal necrolysis (TEN) are rare and potentially life-threatening mucocutaneous blistering diseases, most commonly triggered by medication [1]. Causative medications include antibiotics, antiepileptics, allopurinol, sulfasalazine, and NSAIDs [2, 3]. SJS and TEN are within the same clinical spectrum but with different disease severity, which is determined by the percentage of affected body surface area. Histologically, this needs to be accompanied by subepidermal blisters, apoptotic keratinocytes, and sparse dermal inflammatory infiltrates. The mortality and morbidity risks are significant. An USA-based study showed SJS, SJS/TEN, and TEN to have a mortality of 4.8%, 19.4%, and 14.8%, respectively [4]. The differential diagnosis of SJS/TEN includes erythema multiforme, but also autoimmune bullous diseases, particularly (drug-induced) linear IgA dermatosis (LAD) and paraneoplastic pemphigus (PNP) [5, 6]. Since treatment and prognosis of these diseases differs from SJS/TEN, a complete workup including serologic analysis of desmosomal and hemidesmosomal antibodies is obligatory. On the other hand, several reports have mentioned presence of antibodies against epidermal components in SJS/TEN [7, 8]. We performed extensive serological analysis on 28 confirmed SJS/TEN patients to establish the presence of antibodies against desmosomal and hemidesmosomal proteins.

## 2. Methods

In this retrospective study, the files of all patients admitted to University Medical Centre of Groningen (UMCG) and Martini Hospital Groningen with SJS/TEN were collected for analysis. SJS/TEN was diagnosed based on clinical criteria, e.g., epidermal detachment with erosions and severe mucosal lesions, (suspicion of) a culprit drug, and matching histologic results. Of a total of 28 patients, blood was stored to assess antibodies to skin proteins. There was no need for ethical approval as all the participants in the study had previously consented to share their medical records for research.

Immunoblot (IB), immunoprecipitation (IP), indirect immunofluorescence (IIF), salt-split skin (SSS), ELISA, keratinocyte footprint assay (KFA), and keratinocyte binding assay (KBA).

Routine IB was carried out as described previously [9]. IB reveals the target protein antigens in patients, with molecular weights of 250, 210, and 190 kD. These correlate to desmoplakin I and II, envoplakin, and periplakin. IB was also used to detect plectin antibodies. In selected cases with a high suspicion of PNP, IP was also performed to detect the additional presence of antialpha-2-macroglobulin like 1 (A2ML1) antibodies [10].

IIF using monkey esophagus to detect hemidesmosomal and desmosomal antibodies as a substrate was performed in this study as well as rat bladder to detect antibodies against periplakin and envoplakin. SSS using normal human skin was used to detect hemidesmosomal antibodies. All methods were performed as described previously [11].

ELISAs for the detection of antidesmoglein 1 (Dsg1), antidesmoglein 3 (Dsg3), and the NC16A ectodomain of BP180, BP230 (all MBL, Nagoya, Japan), and IgG autoantibodies were performed according to the manufacturers' instructions. KFA is a technique to detect antilaminin-332 antibodies and is described by us previously [12]. KBA is a very sensitive technique to assess the presence of antibodies against desmosomal proteins [13].

## 3. Results

The cohort consisted of 15 men and 13 women with a mean age of 56 years (Table 1). In most patients, the causative agent was known, and these were in almost all cases common offenders, e.g., antibiotics, antiepileptic medication, and antiretroviral drugs. In most patients, none of the serological tests were positive (Table 2). In two patients, an elevated DSG3 titer was found, suspicious for pemphigus vulgaris although all other tests were negative including KBA. Moreover, in both patients, a biopsy was taken for direct immunofluorescence which showed no extracellular surface binding of immunoglobulins. Three patients had elevated ELISA BP180 NC16a titers, suggesting bullous pemphigoid, but no other tests were positive and in these patients the biopsy for direct immunofluorescence showed no linear binding of immunoglobulins along the basement membrane, excluding a diagnosis of pemphigoid. The three patients with a positive rat bladder and were completely negative for envoplakin and periplakin analysis in the IB as well as the IP, including A2ML1, thereby making a diagnosis of PNP very unlikely. All patients with single positive results were checked for clinical or other laboratory similarities but none were found.

## 4. Discussion

In this SJS/TEN cohort, we extensively characterized the immunological parameters and we found no evidence of circulating pathogenic antibodies against desmosomal and hemidesmosomal proteins. In this respect, it is important not to rely on one single test method since in eight cases, a single false positive test result was found.

There is a resemblance of SJS/TEN with autoimmune blistering diseases, e.g., PNP [6], epidermolysis bullosa acquisita [14], antilaminin-332-pemphigoid [15], and LAD [5]. In fact, these reports in literature showed a significant diagnostic and treatment delay in patients incorrectly diagnosed with SJS/TEN. On the other hand, (false) positive serological tests might hamper the diagnosis and treatment of SJS/TEN. Our results show that in such cases, one can rely on serological analysis to rule out an autoimmune blistering disease, but single results should be interpreted with caution. In three patients, rat bladder testing was positive. A positive rat bladder test might point to a PNP [10] that can have a similar clinical presentation as SJS/TEN with cutaneous erosions and a severe stomatitis. Although one patient (#2) was known with a malignancy, all other tests in these patients, especially IB and IP were negative, making PNP an unlikely diagnosis. In two patients, ELISA testing for DSG and in three patients ELISA testing for BP180 was positive. Based on these results, one cannot rule out pemphigus or bullous pemphigoid. However, all other tests were negative including KBA and salt split skin, which are among the most sensitive and specific serological tests for pemphigus and pemphigoid, respectively [13, 16]. In addition, it is known that ELISA testing might evoke false positive results [16, 17]. Moreover, in all five patients, biopsies for direct immunofluorescence were negative. In none of the 28 patients, the KFA was positive, which implies that laminin-332 can be ruled out as an autoantigen.

There might be several reasons for these single positive serological results in SJS/TEN patients. Keratinocyte destruction by the inflammatory process might result in exposure to self-antigens. This has been shown in amongst other thermal burns and patients with epidermolysis bullosa [18, 19]. In addition, nonpathogenic autoantibodies against desmosomal and hemidesmosomal proteins are present in a low percentage in the general population [20]. Finally, test limitations might result in false positive tests [13].

The pathogenesis of SJS/TEN is only partially understood. It is thought that a neoantigenic-tissue complex is formed with a cytotoxic T-cell response, resulting in massive keratinocyte apoptosis [3]. In addition, studies have shown that in SJS/TEN antibodies against certain desmosomal proteins, e.g., periplakin and desmoplakin [7, 8], could be present although it was questioned whether these antibodies are pathogenic or just a result from the keratinocyte destructing. In our study, we could not confirm the presence of these or other antibodies in a relatively large cohort of SJS/TEN patients. Therefore, it seems unlikely that an antibody-antigen response is involved in SJS/TEN pathogenesis. Although we have done extensive testing for hemidesmosomal and desmosomal proteins, we cannot rule out that an antibody response against other skin proteins might be involved in the pathogenesis of SJS/TEN. Based on direct immunofluorescence, it was suggested that the culprit drug might bind to an intercellular epidermal protein and, therefore, basal keratinocytes are prone to cellular destruction [21]. However, this intercellular protein has never been elucidated and in our hands, IB and IP did not show any clues for other unknown targeted proteins.

In conclusion, the clinical characteristics of SJS/TEN can be very similar to autoimmune bullous diseases, e.g., LAD or PNP. Serological analysis for desmosomal and hemidesmosomal antibodies is reliable to make a certain distinction although one should not rely on one single test, particularly ELISA and rat bladder testing, since false positive results can occur. Moreover, this study also makes it less plausible that antibodies against desmosomal and/or hemidesmosomal components are involved in the pathogenesis of SJS/TEN.

## Figures and Tables

**Table 1:** Patient characteristics.

	Gender	Age	Diagnosis	Causative agent	Comorbidities	Deceased/cause
1	M	69	TEN	Phenytoin	Glioblastoma	Y/glioblastoma
2	F	55	TEN	Phenytoin	Oligo-astrocytoma	Y/brain tumor
3	M	81	TEN	Augmentin	COPD/Parkinson	Y/sputum retention
4	M	60	TEN	Omeprazol, oxybutynin, or simvastatin	CVA	Y/respiratory insufficiency
5	M	60	TEN	Cotrimoxazol	COPD	Y/unknown
6	F	41	SJS/TEN	Paracetamol	NP	N
7	F	2	SJS	Lamictal	Epilepsia	N
8	M	91	TEN	Flucloxacillin, clindamycin, or pantozol	Collum fracture	N
9	F	76	SJS/TEN	Kenacort?	NP	N
10	M	78	TEN	H1N1 vaccination	Urothelial cell carcinoma	Y/unknown
11	F	54	SJS/TEN	Cotrimoxazol	COPD	N
12	M	38	SJS	Nevirapine	HIV	N
13	F	36	SJS	Nevirapine	HIV	N
14	F	62	SJS	Omeprazol?	Lung carcinoma	Y/lung carcinoma
15	M	19	SJS	Nevirapine	HIV	N
16	F	81	SJS/TEN	Zopiclon or amoxicillin	COPD	Y/unknown
17	M	39	TEN	Paracetamol?	NP	N
18	F	77	TEN	Ciprofloxalin	NP	N
19	M	67	SJS	Unknown	NP	N
20	M	55	TEN	Unknown	NP	N
21	F	49	SJS	Carbamazepine	NP	N
22	M	57	TEN	Ceftriaxon	Chronic kidney disease	Y/line sepsis
23	F	60	TEN	Levetiracetam	Melanoma	N
24	M	73	TEN	Vancomycin	Infected hip prosthesis	N
25	F	33	SJS	Lamotrigine	NP	N
26	M	72	TEN	Carboplatin	Lung carcinoma, prostate carcinoma	Y/lung carcinoma
27	F	66	TEN	Sulfasalazine	NP	N
28	M	34	TEN	Lamotrigine	NP	N

SJS = Stevens–Johnson syndrome, TEN = toxic epidermal necrolysis, NP = not present, neg = negative.

**Table 2:** Serological analysis of SJS/TEN patients.

	ME	RB	SSS	IB	IB PNP	IP A2ML1	IB plectin	ELISA DSG1	ELISA DSG3	ELISA NC16a	KFA	IVBA
1	Neg	Neg	Neg	Neg	Neg		Neg	0	0	3	Neg	Neg
2	Neg	Pos	Neg	Neg	Neg	Neg	Neg	0	1	9	Neg	Neg
3	Neg	Neg	Neg	Neg	Neg		Neg	13	1	5	Neg	Neg
4	Neg	Neg	Neg	Neg	Neg		Neg	1	1	0	Neg	Neg
5	Neg	Neg	Neg	Neg	Neg		Neg	0	119	1	Neg	Neg
6	Neg	Neg	Neg	Neg	Neg		Neg	0	1	1	Neg	Neg
7	Neg	Neg	Neg	Neg	Neg		Neg	0	1	1	Neg	Neg
8	Neg	Neg	Neg	Neg	Neg		Neg	0	1	2	Neg	Neg
9	Neg	Neg	Neg	Neg	Neg		Neg	1	1	115/107	Neg	Neg
10	Neg	Neg	Neg	Neg	Neg		Neg	1	1	1	Neg	Neg
11	Neg	Neg	Neg	Neg	Neg		Neg	0	0	2	Neg	Neg
12	Neg	Neg	Neg	Neg	Neg		Neg	7	1	7	Neg	Neg
13	Neg	Neg	Neg	Neg	Neg		Neg	1	3	4	Neg	Neg
14	Neg	Neg	Neg	Neg	Neg		Neg	4	68	1	Neg	Neg
15	Neg	Neg	Neg	Neg	Neg		Neg	8	3	8	Neg	Neg
16	Neg	Pos	Neg	Neg	Neg	Neg	Neg	1	1	1	Neg	Neg
17	Neg	Neg	Neg	Neg	Neg		Neg	1	2	8	Neg	Neg
18	Neg	Neg	Neg	Neg	Neg		Neg	1	1	32/24	Neg	Neg
19	Neg	Pos	Neg	Neg	Neg	Neg	Neg	1	1	1	Neg	Neg
20	Neg	Neg	Neg	Neg	Neg	Neg	Neg	1	0	1	Neg	Neg
21	Neg	Neg	Neg	Neg	Neg		Neg	1	0	1	Neg	Neg
22	Neg	Neg	Neg	Neg	Neg	Neg	Neg	2	1	0	Neg	Neg
23	Neg	Neg	Neg	Neg	Neg		Neg	7	0	0	Neg	Neg
24	Neg	Neg	Neg	Neg	Neg		Neg	2	2	1	Neg	Neg
25	Neg	Neg	Neg	Neg	Neg		Neg	0	1	0	Neg	Neg
26	Neg	Neg	Neg	Neg	Neg		Neg	2	0	1	Neg	Neg
27	Neg	Neg	Neg	Neg	Neg	Neg	Neg	0	0	1	Neg	Neg
28	Neg	Neg	Neg	Neg	Neg	Neg	Neg	0	1	0	Neg	Neg

ME = monkey esophagus, RB = rat bladder, SSS = salt split skin, IB = immunoblot, IP = immunoprecipitation, PNP = paraneoplastic pemphigus, DSG = desmoglein, KFA = keratinocyte footprint assay, IVBA = in vitro binding assay, A2ML1 = *α*_2_-macroglobulin-like protein 1.

## Data Availability

The data used to support the findings of this study are available on request of the corresponding author. The data are not publicly available due to privacy or ethical restrictions.
